# Individual vs. Group Cognitive Behavior Therapy for Anxiety Disorder in Children and Adolescents: A Meta-Analysis of Randomized Controlled Trials

**DOI:** 10.3389/fpsyt.2021.674267

**Published:** 2021-10-20

**Authors:** Tingting Guo, Jing Su, Jiayi Hu, Marianne Aalberg, Yinglin Zhu, Teng Teng, Xinyu Zhou

**Affiliations:** ^1^Department of Psychiatry, The First Affiliated Hospital of Chongqing Medical University, Chongqing, China; ^2^The First Clinical College of Chongqing Medical University, Chongqing, China; ^3^The Second Clinical College of Chongqing Medical University, Chongqing, China; ^4^Division of Mental Health Services, Akershus University Hospital, Lørenskog, Norway; ^5^School of Osteopathic Medicine, Kansas City University of Medicine and Biosciences, Joplin, MO, United States; ^6^Department of Neurology, The First Affiliated Hospital of Chongqing Medical University, Chongqing, China

**Keywords:** anxiety disorder, individual cognitive behavior therapy, group cognitive behavior therapy, children, adolescent, meta-analysis

## Abstract

**Background:** Anxiety disorder is the most prevalent mental disorder in children and adolescents. However, evidence for efficacy and acceptability between individual cognitive behavior therapy (I-CBT) and group cognitive behavior therapy (G-CBT) in anxiety disorders in children and adolescents remains unclear.

**Methods:** Eight electronic databases (PubMed, Embase, Cochrane, Web of Science, CINAHL, PsycINFO, ProQuest, and LILACS) were searched from inception to October 2019. Randomized controlled trials comparing I-CBT with G-CBT for anxiety disorders in children and adolescents were included. The primary outcomes were efficacy (mean change in anxiety symptom scores) at post-treatment and acceptability (all-cause discontinuation). The secondary outcome was remission at post-treatment. Subgroup analyses were also conducted to examine whether the result would be influenced by age, number of treatment sessions, parental involvement, male/female sex, and number of participants.

**Results:** Nine studies were selected in this meta-analysis. The pooled analyses indicated no significant difference between I-CBT and G-CBT for efficacy at post-treatment [standardized mean difference (SMD), −0.14; 95% confidence interval (CI), −0.37 to 0.09], acceptability [odds ratio (OR), 1.30; 95% CI, 0.61–2.77], and remission at post-treatment (OR, 1.15; 95% CI, 0.79–1.66). In the subgroup analysis of age, I-CBT was significantly more effective than G-CBT in adolescents at post-treatment (SMD, −0.77; 95% CI, −1.51 to −0.02), but not in children (SMD, 0.00; 95% CI, −0.02 to 0.20). However, the findings were not materially different from those of the efficacy subgroup analysis of number of treatment sessions, parental involvement, male/female sex, and number of participants.

**Conclusions:** Based on those current evidence, I-CBT was shown to be more beneficial than G-CBT for anxiety disorders in adolescents, but not in children. However, further well-designed clinical studies should be performed to confirm these findings.

**Systematic Review Registration:**http://osf.io/xrjkp, identifier: 10.17605/OSF.IO/XRJKP.

## Background

Anxiety disorders in youth are common, with an estimated lifetime prevalence from 15 to 20%, typically have their onset in childhood or early adolescence (1), and lead to significant psychosocial problems and physical health problems (2). Untreated anxiety disorders in children and adolescents are related to poor functioning and bring a significant risk for psychopathology and dysfunction in their later life (3).

As we know, different treatment interventions are used to treat anxiety, such as medications, psychological treatments, and physical therapy. Currently, several international guidelines recommend that cognitive behavioral therapy (CBT) is the first-line treatment for anxiety disorders in children and adolescents. The National Institute for Health and Care Excellence guideline recommended that CBT focused on treating social anxiety in children and adolescents (4), and the American Academy of Child and Adolescent Psychiatry recommends that CBT be offered to patients in this population with social anxiety, generalized anxiety, separation anxiety, specific phobia, or panic disorder (5). However, the preferred form of CBT was still not clear (6). Individual CBT (I-CBT) and group CBT (G-CBT) are the two most common forms of treatment of anxiety disorders in children and adolescents. Some previous studies reported that G-CBT was likely to offer more opportunities for normalization, positive peer modeling, reinforcement, social support, and exposure to social situations (7). Meanwhile, G-CBT is more cost-effective, so it can save medical resources (8). On the contrary, some trials showed that I-CBT would offer more opportunities for individualization of treatment to address the specific needs of each patient, and avoidant behavior may be more readily addressed (9). Consequently, the question on how to choose the form of CBT for the treatment of anxiety disorder in young people remains controversial.

Thus, to address the abovementioned issue, we designed a meta-analysis of randomized controlled trials to compare the efficacy and acceptability of I-CBT and G-CBT for anxiety disorder in children and adolescents.

## Methods

### Data Sources and Searches

This study was reported by using PRISMA guidelines (Supplementary Table 1) (10). Seven relevant published electronic databases (PubMed, Embase, Cochrane, Web of Science, CINAHL, PsycINFO, and LILACS) and one unpublished database (ProQuest Dissertation Abstracts) were searched for the trials from the date of database inception to October 2019 using the following keywords: (“anxiety” OR “anxious” OR “phobic” OR “fear^*^” OR “phobia^*^”) and (“adolesc^*^” OR “child^*^” OR “boy^*^” OR “girl^*^” OR “juvenil^*^” OR “minors” OR “paediatri^*^” OR “pediatri^*^” OR “pubescen^*^” OR “school^*^” OR “student^*^” OR “teen^*^” OR “young”) and (“behavio^*^” OR “cogniti^*^” OR “CBT”) and (“individual” OR “I-CBT,” and “group” OR “G-CBT”). The details of the systematic search terms and strategies are displayed in Supplementary Table 2. Furthermore, to identify additional eligible randomized controlled trials (RCTs) and reviews, the reference lists of relevant studies were scanned, and we also contacted all relevant authors in cases of incomplete information. No language restrictions were applied to the search.

### Study Selection

Any RCTs that compared the efficacy and acceptability of the CBT delivery formats of individual (I-CBT) with group (G-CBT) in the treatment of anxiety disorders in children or adolescents with or without parents were identified. The titles and abstracts identified from the search strategies were independently examined by two reviewers (TG and JS). If both reviewers judged the trial as not having met the eligibility criteria, it was excluded. Then, we obtained the full text of all remaining articles and determined whether to include them according to the inclusion criteria. Any disagreements were resolved by a third reviewer (XZ). The inclusion criteria were as follows: (1) any RCTs, including crossover trials and cluster randomized trials, were included, but trials whose duration of treatment was <6 weeks and trials whose number of sessions was < six sessions were excluded; (2) children and adolescents under the age of 18 with a primary diagnosis of anxiety disorder according to standardized diagnostic criteria, e.g., the Diagnostic and Statistical Manual of Mental Disorders (DSM) (11) and the Anxiety Disorders Interview Schedule (ADIS) (12) were included; and (3) any RCTs that compared the efficacy of I-CBT and G-CBT for anxiety disorders in children and adolescents were included.

### Outcome Measures

To evaluate the effect of CBT, the mean change scores of the anxiety rating scale from baseline to post-treatment (efficacy) and the proportion of patients who discontinued treatment for any reason up to post-treatment (acceptability) were defined as primary outcomes. When anxiety symptoms had been measured with more than one standardized rating scale, we used a pre-defined hierarchy based on psychometric properties, frequency of use in children and adolescents, and consistency of use across the included trials.

The second efficacy outcome was remission rate, which is measured by the proportion of participants who achieved a reduction of 50% or more in anxiety rating score or who scored much or very much improved on the anxiety rating scales (e.g., SPAI-C total score <18 and ADIS-IV-C/P total score <4) (13, 14).

### Data Extraction and Quality Assessment

Two independent researchers (JS and TG) extracted the data and assessed the risk of bias. The researchers extracted the key characteristics of studies using a standardized data abstraction form, which included titles, diagnostic criteria, number of patients, treatment comparators, age range, man/female, treatment duration, number of sessions, parental involvement, and measure outcomes. We also assessed the risk of bias in studies using the Risk of Bias Tool from the Cochrane Handbook 5.0.1. Any disagreements were resolved by a third researcher (XZ).

### Statistical Analysis

We performed a meta-analysis with Review Manager 5.3.5 to compare the relative efficacy and acceptability. We used a random-effects model to perform the meta-analyses by synthesizing studies that compared the same interventions (15). The effect sizes were expressed using standardized mean difference (SMD) with 95% confidence intervals (CIs) for continuous outcomes and odds ratios (OR) with 95% CIs for discontinuous outcomes (16). The heterogeneity of treatment effects across studies was assessed by *I*^2^ and the Q-statistic test (17). Funnel plots were conducted to detect a possible publication bias, and Egger's regression asymmetry test was conducted to conclude whether there was a significant publication bias (18). We also conducted subgroup analyses to examine whether the result would be influenced by parental involvement (with *vs*. without), number of treatment sessions (<12 vs. ≥12 sessions), age (children vs. adolescents), male/female sex (<1 vs. ≥1), number of participants (<100 vs. ≥100), and publication years (<2010 vs. ≥2010). In addition, we did a subgroup analysis of parental involvement in children or adolescents.

## Results

### Study Selection and Characteristics

**Figure 1:**
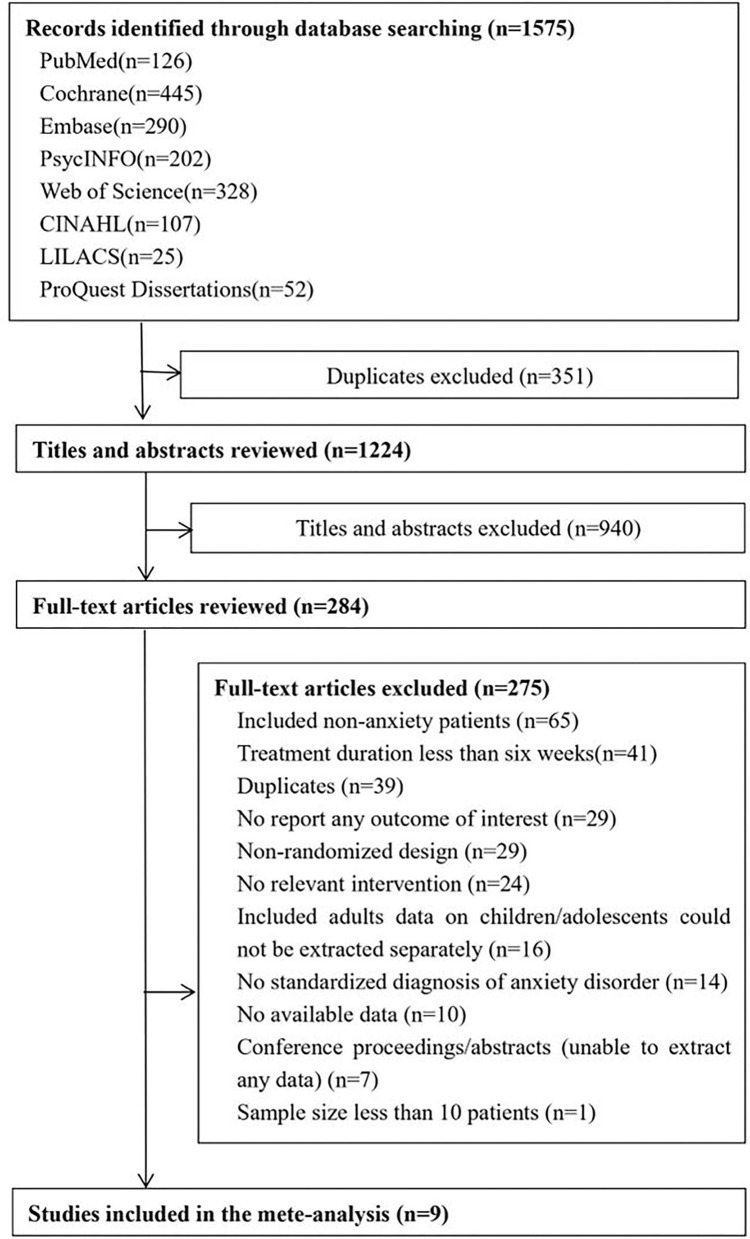
Flow chart of the study selection.

After searching seven electronic databases, we identified 1,575 potentially relevant studies. Of these, 351 duplicates were excluded and 940 documents were excluded because their titles and abstracts met the exclusion criteria. Then, 284 full-text articles were identified for review. The interrater reliability of the two independent reviewers was 0.781 (Cohen's kappa). In total, nine RCTs (13, 14, 19–25) with a total of 871 participants and published between 2000 and 2018 were included in this meta-analysis (Figure 1). Overall, there were 349 participants in the I-CBT group and 355 participants in the G-CBT group, and there were 167 participants in the control conditions (waitlist, *n* = 107; psychological placebo, *n* = 60).

The clinical characteristics of each trial are summarized in Table 1. The mean age of the participants was 11.49 years (SD 2.19), and about half of the sample population were female (44.89%). The mean sample size was 96.78 participants (SD 56.41). The median duration of the acute treatment was 12 weeks (range, 6–18), and the median number of sessions was 12 (range, 10–18). All the included studies investigated participants diagnosed with anxiety disorders and used both individual and group format CBT interventions.

**Table 1:** Characteristics of included studies.

**References**	**Type of anxiety**	**Diagnostic criteria**	**No. of patients**	**Treatment comparators**	**Age range, y (mean)**	**M/F**	**Setting**	**Treatment duration, wk**	**No. of sessions**	**Parental involvement**	Co-medication*	**Outcome measure**	**Baseline severity, mean (SD)**	**Primary efficacy (post-treatment)**	**Risk of bias**
de Groot et al. (14)	GAD, SAD, Sep, SP	ADIS-IV C/P	29	I-CBT = 14 Vs. G-CBT = 15	7–12 (8.9)	1.90	University clinic	12	12 vs. 12 vs. NA	Yes	NA	SCAS	33.79 (16.87)	−10.21 vs. −6.93	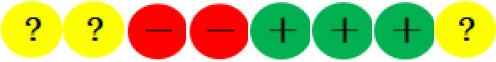
Flannery-Schroeder and Kendall (22)	GAD, Sep, SAD	ADIS-IV C/P	45	I-CBT = 18 vs. G-CBT = 13 vs. WL = 14	8–14 (NA)	1.06	University clinic	18	18 vs. 18 vs. NA	No	NA	RCMAS	52.77 (11.58)	−10.9 vs. −8.4 vs. −2	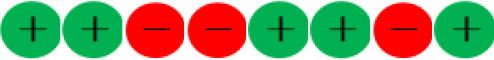
Herbert et al. (13)	SAD	ADIS-IV C	73	I-CBT = 24 vs. G-CBT = 23 vs. PBO = 26	13–17 (14.7)	0.78	University clinic	12	12 vs. 12 vs. 12	No	Yes (*N* = 11, 15.1%)	SPAI	39.68 (16.82)	−10.8 vs. −3.46 vs. −4.53	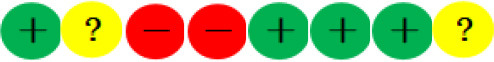
Ingul et al. (20)	SAD	ADIS-IV C	128	I-CBT = 36 vs. G-CBT = 58 vs. PBO = 34	13–16 (14.5)	0.78	Not stated	12	12 vs. 10 vs. 10	No	Not stated	SCARED	24.18 (13.44)	−13.27 vs. 2.56 vs. −0.26	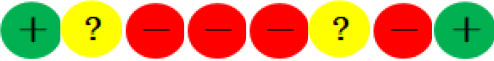
Liber et al. (19)	Sep, GAD, SAD, SP	ADIS-IV C/P	127	I-CBT = 65 vs. G-CBT = 62	8–12 (10.0)	1.27	University clinic	17	10 vs. 10 vs. NA	Yes	Yes (*N* = 5, 3.9%)	MASC	51.13 (18.37)	−13.91 vs. −14.43	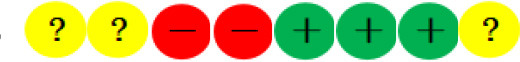
Manassis et al. (25)	GAD, Sep, SP, SAD, PD	DICA-R	86	I-CBT = 43 vs. G-CBT = 43	8–12 (10.0)	1.17	Outpatient clinic	12	12 vs. 12 vs. NA	Yes	Yes (*N* = 8, 9.3%)	MASC	52.82 (10.19)	−3.95 vs. −1.83	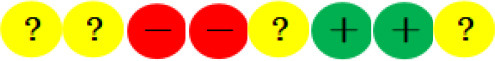
Muris et al. (24)	Sep, GAD, SAD	DISC 2.3	36	I-CBT = 17 vs. G-CBT = 19	8–12 (9.9)	0.33	School	6	12 vs. 12 vs. NA	No	Yes (*N* = 1, 2.7%)	STAIC	44.08 (7.61)	−7 vs. −11.4	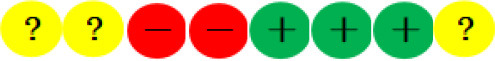
Villabø et al. (21)	SAD, Sep, GAD	DSM-IV-TR	165	I-CBT = 55 vs. G-CBT = 55 vs. WL = 55	7–12 (10.5)	1.20	Community clinic	12	14 vs. 14 vs. NA	No	NA	MASC	57.63 (10.86)	−8.69 vs. −8.83 vs. −6.02	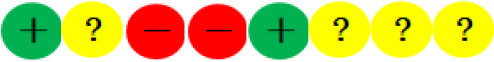
Wergeland et al. (23)	Sep, SAD, GAD	ADIS-IV C/P	182	I-CBT = 77 vs. G-CBT = 67 vs. WL = 8	8–15 (11.5)	0.90	Community clinic	12	10 vs. 10 vs. NA	Yes	Yes (*N* = 11, 6.0%)	SCAS	36.09 (16.72)	−8.79 vs. −8.95	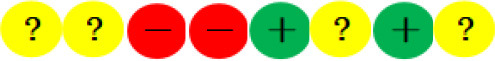

*GAD, generalized anxiety disorder; SAD, social anxiety disorder/social phobia; Sep, separation anxiety disorder; SP, specific/simple phobia; PD, panic disorder; ADIS, Anxiety Disorders Interview Schedule for DSM-4; DISC, Diagnostic Interview Schedule for Children; DICA, Diagnostic Interview for Children and Adolescents-Revised; DSM, Diagnostic and Statistical Manual of Mental Disorders; M/F, man/female; SCAS, Spence Children's Anxiety Scale; RCMAS, Revised Children's Manifest Anxiety Scale; SPAI, Social Phobia Anxiety Inventory for Children; SCARED, Screen for Child Anxiety Related Emotional Disorders; MASC, Multidimensional Anxiety Scale for Children; STAIC, State–Trait Anxiety Inventory for Children; the risk of bias were as follows: random sequence generation, allocation concealment, blinding of participants and personnel, blinding of outcome assessment, incomplete outcome data, selective reporting, other bias and total risk, I-CBT, individual cognitive behavioral therapy; G-CBT, group cognitive behavioral therapy; WL, waitlist; PBO, psychological placebo*.

* *Co-medication interventions included psychotropic medications, such as methylphenidate and serotonin reuptake inhibitors*.

*−: high risk of bias, +: low risk of bias, ?: unclear risk of bias*.

### Quality Assessment

There were four studies with a low risk of bias owing to random sequence generation. Only one study reported a low risk of bias owing to allocation concealment. All of the studies reported a high risk of bias owing to blinding of participants and personnel and blinding of outcome assessment. Only one study reported a high risk of bias owing to incomplete outcome data. One study reported a low risk of bias owing to selective reporting. There were three studies with a low risk of bias owing to random other biases (Table 1). The funnel plot for efficacy at post-treatment and acceptability can be seen in Supplementary Figure 1. The Egger tests indicated a publication bias in efficacy at post-treatment (*t* = 0.69, *p* = 0.502) and acceptability (*t* = 2.19, *p* = 0.080).

### Primary and Secondary Outcomes

**Figure 2:**
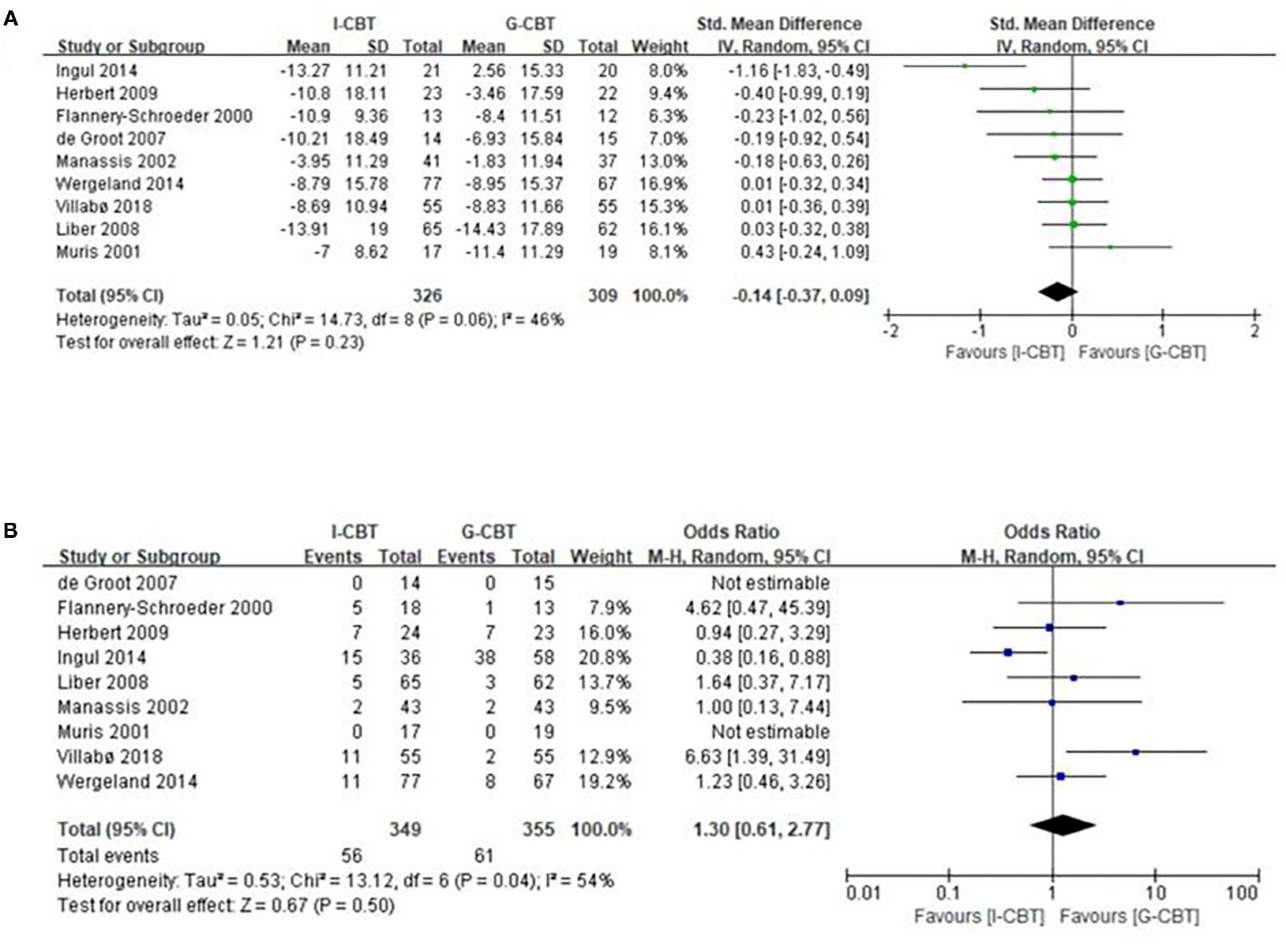
Meta-analysis of primary efficacy outcome. **(A)** Forest plot of standardized mean difference (SMD) for changed scores in anxiety disorder rating scales in the comparison between individual cognitive behavior therapy (I-CBT) and group cognitive behavior therapy (G-CBT). **(B)** Forest plot of odds ratios (with 95% confidence intervals) of discontinuance for any reason in the comparison between I-CBT and G-CBT.

For the primary outcome of efficacy at post-treatment, the overall pooled effect size indicated no significant difference between the I-CBT group and the G-CBT group with SMD of −0.14 (95% CI, −0.37 to 0.09, *p* = 0.23) and moderate heterogeneity (*I*^2^ = 46%, *p* = 0.06; Figure 2A). For the acceptability outcome, there was no significant difference between the I-CBT group and the G-CBT group with OR of 1.30 (95% CI, 0.61–2.77, *p* = 0.50) and high heterogeneity (*I*^2^ = 54%, *p* = 0.04; Figure 2B).

**Figure 3:**
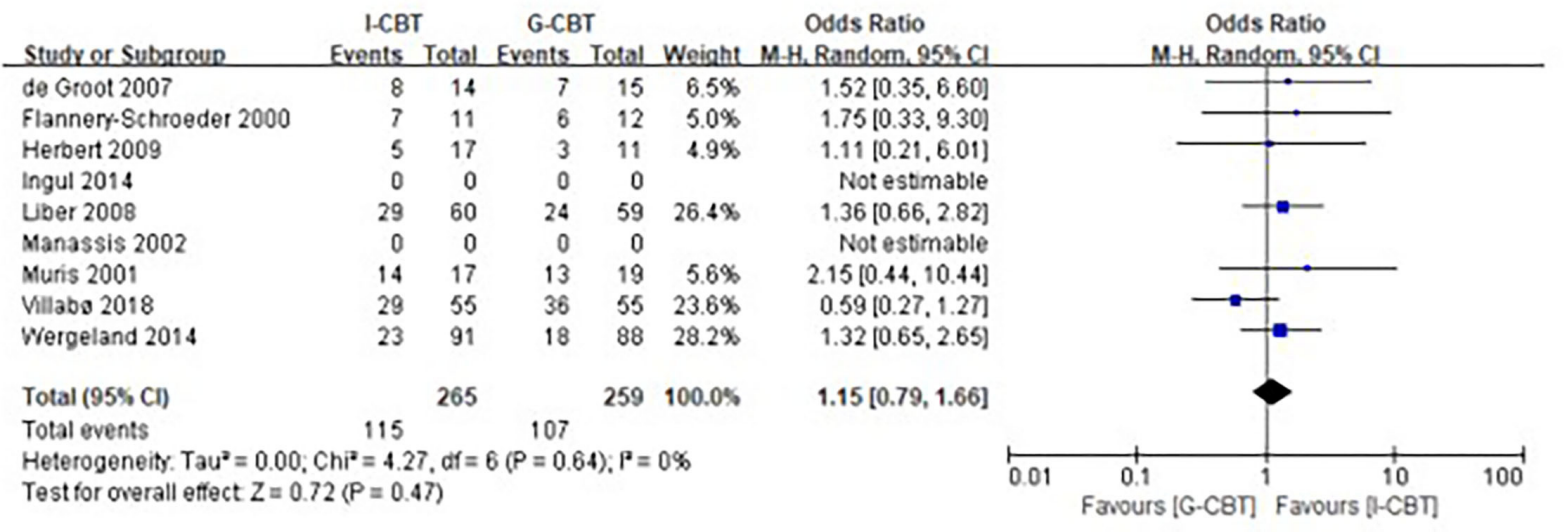
Meta-analysis of the rate of remission. Forest plot of odds ratios (with 95% confidence intervals) of remission at post-treatment in the comparison between individual cognitive behavior therapy and group cognitive behavior therapy.

For the rate of remission at post-treatment, there was also no statistical difference between the I-CBT group and the G-CBT group with OR of 1.15 (95% CI, 0.79–1.66, *p* = 0.47) and low heterogeneity (*I*^2^ = 0%, *p* = 0.64; Figure 3).

### Subgroup Analysis

**Figure 4:**
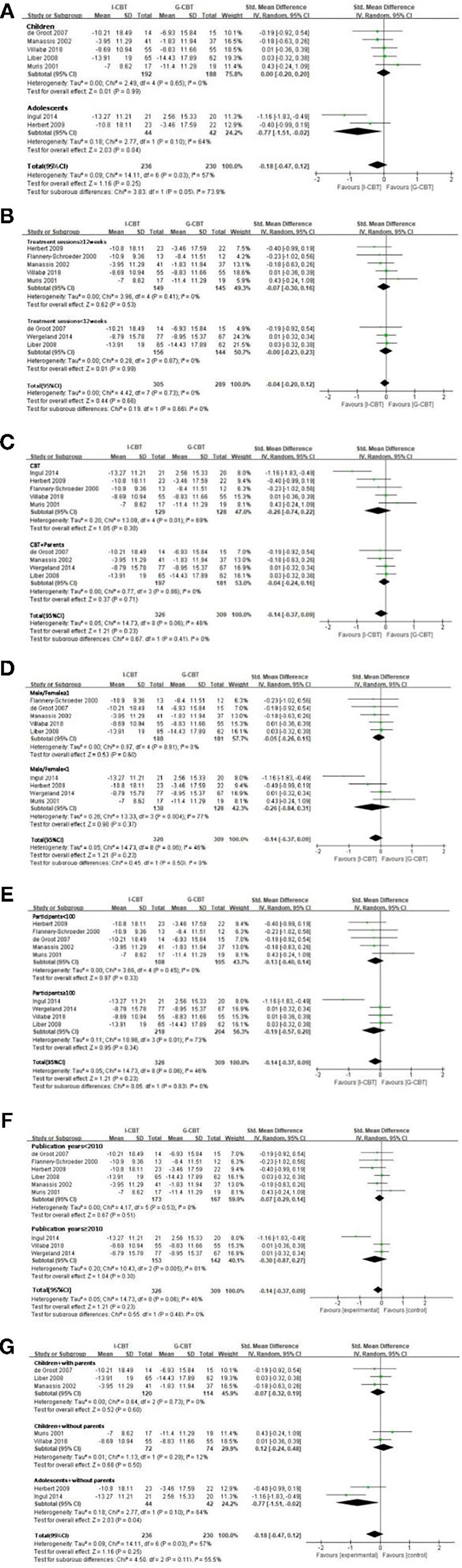
Subgroup analyses of primary efficacy outcome. **(A)** Forest plot of standardized mean difference (SMD) for changed scores in anxiety disorder rating scales for age subgroup. **(B)** Forest plot of SMD for changed scores in anxiety disorder rating scales for treatment session subgroup. **(C)** Forest plot of SMD for changed scores in anxiety disorder rating scales for parental involvement subgroup. **(D)** Forest plot of SMD for changed scores in anxiety disorder rating scales for male/female subgroups. **(E)** Forest plot of SMD for changed scores in anxiety disorder rating scales for number of participants subgroup. **(F)** Forest plot of SMD for changed scores in anxiety disorder rating scales for publication years subgroup. **(G)** Forest plot of SMD for changed scores in anxiety disorder rating scales for parental involvement in children or adolescent subgroup.

We also studied the effect of several potential moderator variables, including age, for the primary efficacy outcome in subgroup analyses. For the subgroup analysis of age (Figure 4A), I-CBT was significantly more beneficial than G-CBT (SMD, −0.77; 95% CI, −1.51 to −0.02; *p* = 0.04) in studies with adolescents (13–17 years old). However, in studies with children (7–12 years old), I-CBT did not differ significantly from G-CBT (SMD, 0.00; 95% CI, −0.20 to 0.20; *p* = 0.99) at post-treatment. However, the findings were not materially different from those of the efficacy analysis for the subgroup of number of treatment sessions (<12 vs. ≥12 sessions, Figure 4B), parental involvement (with vs. without, Figure 4C), male/female sex (<1 vs. ≥1, Figure 4D), number of participants (<100 vs. ≥100, Figure 4E), and publication years (<2010 vs. ≥2010, Figure 4F). In addition, we did a subgroup analysis of parental involvement in children or adolescents. In the child group, there was no material difference between with parents and without parents. However, none of the parents are involved in the adolescent group (Figure 4G).

## Discussion

To our knowledge, this is the first meta-analysis that synthesized RCTs on comparing I-CBT with G-CBT for children and adolescents with anxiety disorders. According to our research, there were no significant differences between I-CBT and G-CBT in terms of anxiety symptom reduction and anxiety disorder diagnosis remission at post-treatment. However, the subgroup analysis showed that I-CBT was significantly more effective than G-CBT in the adolescent population.

In a meta-analysis about CBT in the treatment of anxiety disorders in children and adolescents, it was found that CBT was more effective than no treatment in reducing anxiety symptoms in children and adolescents, but a difference in outcome was noted between I-CBT and G-CBT from indirect evidence (6). However, in a meta-analysis of anxiety disorder in an adult population, I-CBT was more effective than G-CBT (26). This difference between juvenile and adult can be explained by the following reasons: First of all, this study only compared the efficacy of I-CBT and G-CBT with psychological placebo, respectively, and found that I-CBT was superior to psychological placebo, but G-CBT not. Secondly, patients may have to wait longer to begin the treatment than in G-CBT because it takes time to assemble a group. There was also less flexibility about when the sessions can be scheduled in G-CBT, which may lead to less complete attendance than in individual treatment (27). Besides this, I-CBT can offer an individualized therapeutic regimen for anxiety patients, especially under the potential presence of comorbid or multi-morbid mental and physical health disorders (28).

In the subgroup analysis, I-CBT was significantly more effective than G-CBT in adolescents (13–17 years old), but not in children (7–12 years old). The results can be explained by two possible reasons: First, there is rapid cognitive development from childhood to adolescence (29), and I-CBT may change the specific cognitive factors of anxiety disorders in adolescents more easily than in children. Second, the participants involved in the two adolescent subgroup studies had high rates of social anxiety disorder, which had a better efficacy in individual format (13, 20). Similar findings are reported in studies of adults (28, 30), suggesting that cognitive developmental factors and high rates of social anxiety disorder, as observed in these studies, may in part explain the greater benefit of individual treatment format. A recent study reported that about half of adolescents who retained their anxiety diagnoses at post-treatment lost the diagnoses at long-term follow-up (31). Some researchers explained it as the delayed treatment effect, which stems from the acquired skills among adolescent and parents (32). This is consistent with the results of the studies that we included (13, 20, 22). However, since some subjects did not complete a systematic follow-up, there is still some controversy in this result, which needs further verification. A study said I-CBT might be the better treatment for school-aged children with anxiety disorders (33). Our inclusion criteria include school-age children, while this issue was not discussed in depth in subsequent studies. Based on the results, so far there is no statistically significant difference in children between I-CBT and G-CBT. The subgroup analysis of the number of treatment sessions (≥12 vs. <12 weeks) showed that I-CBT was not significantly more effective than G-CBT. The National Health Service in the United Kingdom recommends that CBT is delivered with 5–20 weekly or biweekly sessions of 30–60 min. In the studies included in the present analyses, most treatments consisted of 10 or 12 sessions. Although some researchers reported that longer treatment durations of CBT may result in better efficacy (34), differences in efficacy in long or short treatment duration were not observed. Regarding the subgroup analysis of parental involvement, it was found to be not associated with better treatment outcome. A previous study revealed that CBT with parental involvement increased the treatment efficacy, especially in young children who have at least one anxious parent (35). However, the effect of parental involvement may increase the efficacy in I-CBT or G-CBT in a similar manner. Although there were no significant differences among subgroups with or without parental involvement, there are reports in the literature that showed a strong family component in childhood anxiety (36). Parenting behaviors, emotional openness, and the type of secure attachment may influence the prognosis (33, 36, 37). Walter et al. considered family-directed interventions to be a supplement in individual treatment (5).

The subgroup analysis of sex ratio showed that there was no significant difference between I-CBT and G-CBT. The same result was reported in a meta-analysis of adult anxiety disorder (38). A German study of adolescent psychological problems came to the same conclusion that gender difference did not affect the results of the study (39). Our study did not divide the subgroups into different anxiety types because the diagnosis of subjects in the included literature is very complex, and there are boundaries and overlap in the division of different anxiety types (40). These factors all have different degrees of influence on the efficacy, which is not conducive to our analysis.

I-CBT and G-CBT did not differ in terms of acceptability outcomes and all-cause discontinuation. However, I-CBT and G-CBT had relatively high withdrawal rates of the participants at the end of the RCTs. This finding may result from the fact that acceptability in psychotherapy is more related to efficacy rather than tolerability, and adverse events were rarely reported in clinical trials of psychotherapy (41). Carl R. Rogers believes empathy, genuineness, and warmth are the fundamental qualities of being a qualified therapist (42). Multiple subjects means that a therapist may have less chance to individually design a plan for each subject or build further relationship (33, 43). Hence, there are huge advantages in I-CBT to stay coherent with the individual personality and family dynamic of each subject. Group therapy means social bonding. Children and adolescents have imperfect social relationships. Especially in children, they gradually notice the social differences between each other (44), which would not happen in I-CBT. Meanwhile, negative peer modeling and social distractions may make the therapist spend a lot of time on non-therapeutic procedures (22). I-CBT would be a good choice for children with insecure attachment styles (45), while children who need more positive role models may choose G-CBT (46).

### Limitation

Some limitations in our meta-analysis warrant mention. First, although we have conducted a systematic and comprehensive search, these clinical findings should be interpreted with caution as the number of included studies was relatively small, and the heterogeneity was relatively high, with an uncertainty around these estimates in most of the subgroup results, so further clinical trials are needed to provide evidence. In addition, the overall quality of the included studies was low, and most studies were published more than 10 years ago. Second, except for two studies including participants with social anxiety, most of the studies concentrated on mixed anxiety disorders, which may increase the heterogeneity in the meta-analysis (47), and did not consider anxiety disorders and subtypes in the international classification and diagnostic system. Third, all the included studies involved self-report scales of anxiety symptoms of children. When using self-reported ratings, there may be systematic biases across groups, especially in psychotherapy, as the participants knew the conditions of the groups to which they have been assigned. Fourth, the data of the follow-up in this study were not sufficient, and the long-term follow-up effect of I-CBT and G-CBT needs to be further studied. Fifth, because of the different therapy methods between individual and group settings, it is difficult to control the blinding in the RCTs in this study (48). Furthermore, the relationship between patient and therapist may be stronger in individual setting, while in group setting other mechanisms are more important, such as cohesion and social support (49). These different mechanisms of group and individual settings could not be controlled in this study, which may result in different effect sizes (50).

## Conclusion

This meta-analysis suggests that I-CBT was shown to be more beneficial than G-CBT for anxiety disorders in adolescents, but not in children. However, clinicians should interpret these findings carefully.

## Data Availability Statement

The original contributions presented in the study are included in the article/Supplementary Material, further inquiries can be directed to the corresponding authors.

## Author Contributions

XZ and TT designed the experiments. TG, JS, JH, MA, and YZ conducted the experiments. TG, JS, and JH analyzed the data and drafted the manuscript. All authors contributed to the article approved the submitted version.

## Funding

This study was funded by the Science and Technology Research Project of Chongqing Education Commission (KJQN201800415) and the High-end R&D Talent Support Project of Chongqing Science and Technology Bureau (cstc2018kjcxljrc0038).

## Conflict of Interest

The authors declare that the research was conducted in the absence of any commercial or financial relationships that could be construed as a potential conflict of interest.

## Publisher's Note

All claims expressed in this article are solely those of the authors and do not necessarily represent those of their affiliated organizations, or those of the publisher, the editors and the reviewers. Any product that may be evaluated in this article, or claim that may be made by its manufacturer, is not guaranteed or endorsed by the publisher.

## Abbreviations

CBT: cognitive behavioral therapy
I-CBT: individual cognitive behavior therapy
G-CBT: group cognitive behavior therapy
NICE: The National Institute for Health and Care Excellence
AACAP: American Academy of Child and Adolescent Psychiatry
SMD: standardized mean difference
CI: confidence interval
OR: odds ratio
RCTs: randomized controlled trials
ADIS: Anxiety Disorders Interview Schedule.
