# Supplementary material for: Individual vs. Group Cognitive Behavior Therapy for Anxiety Disorder in Children and Adolescents: A Meta-Analysis of Randomized Controlled Trials
Source: Front Psychiatry. 2021 Oct 20;12:674267. doi: 10.3389/fpsyt.2021.674267 (PMC8564073; doi:10.3389/fpsyt.2021.674267)

**Supplementary Content**

**Table S1**

PRISMA checklist

**Table S2**

Literature search report: Cochrane (445), Embase (290), ProQuest (50), PsycINFO (324), PubMed (126), Web of Science (328),CINAHL(81), LILACS(25)

**Figure S1**

Funnel plot for primary efficacy outcomes: the standardized mean difference for changed scores in anxiety disorder rating scales at post-treatment **(A)** and acceptability **(B)**.

**Table S1. PRISMA checklist**

| **Section/topic** | **#** | **Checklist item** | **Reported on page #** |
| --- | --- | --- | --- |
| **TITLE** | | |  |
| Title | 1 | Identify the report as a systematic review, meta-analysis, or both. | 1 |
| **ABSTRACT** | | |  |
| Structured summary | 2 | Provide a structured summary including, as applicable: background; objectives; data sources; study eligibility criteria, participants, and interventions; study appraisal and synthesis methods; results; limitations; conclusions and implications of key findings; systematic review registration number. | 2 |
| **INTRODUCTION** | | |  |
| Rationale | 3 | Describe the rationale for the review in the context of what is already known. | 3 |
| Objectives | 4 | Provide an explicit statement of questions being addressed with reference to participants, interventions, comparisons, outcomes, and study design (PICOS). | 3 |
| **METHODS** | | |  |
| Protocol and registration | 5 | Indicate if a review protocol exists, if and where it can be accessed (e.g., Web address), and, if available, provide registration information including registration number. | NA |
| Eligibility criteria | 6 | Specify study characteristics (e.g., PICOS, length of follow-up) and report characteristics (e.g., years considered, language, publication status) used as criteria for eligibility, giving rationale. | 4-5 |
| Information sources | 7 | Describe all information sources (e.g., databases with dates of coverage, contact with study authors to identify additional studies) in the search and date last searched. | 4 |
| Search | 8 | Present full electronic search strategy for at least one database, including any limits used, such that it could be repeated. | 4 |
| Study selection | 9 | State the process for selecting studies (i.e., screening, eligibility, included in systematic review, and, if applicable, included in the meta-analysis). | 4 |
| Data collection process | 10 | Describe method of data extraction from reports (e.g., piloted forms, independently, in duplicate) and any processes for obtaining and confirming data from investigators. | 6 |
| Data items | 11 | List and define all variables for which data were sought (e.g., PICOS, funding sources) and any assumptions and simplifications made. | 5 |
| Risk of bias in individual studies | 12 | Describe methods used for assessing risk of bias of individual studies (including specification of whether this was done at the study or outcome level), and how this information is to be used in any data synthesis. | 6 |
| Summary measures | 13 | State the principal summary measures (e.g., risk ratio, difference in means). | 5 |
| Synthesis of results | 14 | Describe the methods of handling data and combining results of studies, if done, including measures of consistency (e.g., I^2^) for each meta-analysis. | 6 |

Page 1 of 2

| **Section/topic** | **#** | **Checklist item** | **Reported on page #** |
| --- | --- | --- | --- |
| Risk of bias across studies | 15 | Specify any assessment of risk of bias that may affect the cumulative evidence (e.g., publication bias, selective reporting within studies). | 8 |
| Additional analyses | 16 | Describe methods of additional analyses (e.g., sensitivity or subgroup analyses, meta-regression), if done, indicating which were pre-specified. | 7 |
| **RESULTS** | | |  |
| Study selection | 17 | Give numbers of studies screened, assessed for eligibility, and included in the review, with reasons for exclusions at each stage, ideally with a flow diagram. | 7 |
| Study characteristics | 18 | For each study, present characteristics for which data were extracted (e.g., study size, PICOS, follow-up period) and provide the citations. | 7 |
| Risk of bias within studies | 19 | Present data on risk of bias of each study and, if available, any outcome level assessment (see item 12). | 8 |
| Results of individual studies | 20 | For all outcomes considered (benefits or harms), present, for each study: (a) simple summary data for each intervention group (b) effect estimates and confidence intervals, ideally with a forest plot. | 8 |
| Synthesis of results | 21 | Present results of each meta-analysis done, including confidence intervals and measures of consistency. | 8 |
| Risk of bias across studies | 22 | Present results of any assessment of risk of bias across studies (see Item 15). | 8 |
| Additional analysis | 23 | Give results of additional analyses, if done (e.g., sensitivity or subgroup analyses, meta-regression [see Item 16]). | 9 |
| **DISCUSSION** | | |  |
| Summary of evidence | 24 | Summarize the main findings including the strength of evidence for each main outcome; consider their relevance to key groups (e.g., healthcare providers, users, and policy makers). | 9 |
| Limitations | 25 | Discuss limitations at study and outcome level (e.g., risk of bias), and at review-level (e.g., incomplete retrieval of identified research, reporting bias). | 12 |
| Conclusions | 26 | Provide a general interpretation of the results in the context of other evidence, and implications for future research. | 12 |
| **FUNDING** | | |  |
| Funding | 27 | Describe sources of funding for the systematic review and other support (e.g., supply of data); role of funders for the systematic review. | 13 |

*From:*  Moher D, Liberati A, Tetzlaff J, Altman DG, The PRISMA Group (2009). Preferred Reporting Items for Systematic Reviews and Meta-Analyses: The PRISMA Statement. PLoS Med 6(7): e1000097. doi:10.1371/journal.pmed1000097

**Table S2. Search Strategy and Results**

Number of citations by each database and trial register searched

| **Databases:** | **Citations** |
| --- | --- |
| PubMed | 126 |
| Cochrane | 445 |
| Embase | 290 |
| PsycINFO | 202 |
| Web of Science | 328 |
| CINAHL | 107 |
| LILACS | 25 |
| ProQuest Dissertations | 52 |
| Total (databases) | 1575 |

**Full search strategy for each database**

**PubMed**

#1 behavio*[Title/Abstract] AND cogniti*[Title/Abstract] AND therapy[Title/Abstract]

#2 CBT[Title/Abstract] OR “Cognitive behavior therapy”[Title/Abstract] OR “Cognitive behavioral therapy”[Title/Abstract] OR “Cognitive behaviour therapy”[Title/Abstract] OR “Cognitive behavioural therapy”[Title/Abstract] OR “Cognitive-behavioural therapy”[Title/Abstract] OR “Cognitive-behavioual therapy”[Title/Abstract]

#3 #1 OR #2

#4 “group ”[Title/Abstract] AND “individual ”[Title/Abstract]

#5 #3 AND #4

#6 "Anxiety Disorders"[Mesh]

#7 anxiety[Title/Abstract] OR anxious[Title/Abstract] OR phobic[Title/Abstract] OR fear[Title/Abstract] OR fears[Title/Abstract] OR phobia[Title/Abstract] OR phobias[Title/Abstract] OR “panic disorder*”[Title/Abstract] OR “overanxious disorder*”[Title/Abstract] OR “avoidant disorder*”[Title/Abstract] OR agoraphobia[Title/Abstract] OR “selective mutism”[Title/Abstract] OR “panic attack speciﬁer”[Title/Abstract] OR “combat disorder*”[Title/Abstract] OR “mixed disorder*”[Title/Abstract] OR neurosis[Title/Abstract] OR neuroses[Title/Abstract] OR neurotic[Title/Abstract] OR “school refusal”[Title/Abstract]

#8 #6 OR #7

#9 adolesc*[Title/Abstract] OR child*[Title/Abstract] OR boy*[Title/Abstract] OR girl*[Title/Abstract] OR juvenil*[Title/Abstract] OR minors[Title/Abstract] OR paediatri*[Title/Abstract] OR pediatri*[Title/Abstract] OR pubescen*[Title/Abstract] OR school*[Title/Abstract] OR student*[Title/Abstract] OR teen*[Title/Abstract] OR young[Title/Abstract] OR youth*[Title/Abstract] OR class*[Title/Abstract] OR preschool[Title/Abstract] OR pre-school[Title/Abstract]

#10 "Child"[Majr] OR "Adolescent"[Majr]

#11 #9 OR #10

#12 #5 AND #8 AND #11

**Cochrane**

#1 MeSH descriptor: [Cognitive Behavioral Therapy] explode all trees

#2 (behavio*):ti AND (cogniti*):ti AND (therapy):ti OR (CBT):ti (Word variations have been searched)

#3 (behavio*):ab AND (cogniti*):ab AND (therapy):ab OR (CBT):ab (Word variations have been searched)

#4 #1 OR #2 OR #3

#5 (individual AND group):ab(Word variations have been searched)

#6 (individual AND group):ti (Word variations have been searched)

#7 #5 OR #6

#8 #4 AND #7

#9 MeSH descriptor: [Anxiety Disorders] explode all trees

#10 (anxiety or anxious or phobic or fear or fears or phobia or phobias or "panic disorder*" or "overanxious disorder*" or "avoidant disorder*" or agoraphobia or "selective mutism" or "panic attack speciﬁer" or "combat disorder*" or "mixed disorder*" or neurosis or neuroses or neurotic or "school refusal"):ti (Word variations have been searched)

#11 (anxiety or anxious or phobic or fear or fears or phobia or phobias or "panic disorder*" or "overanxious disorder*" or "avoidant disorder*" or agoraphobia or "selective mutism" or "panic attack speciﬁer" or "combat disorder*" or "mixed disorder*" or neurosis or neuroses or neurotic or "school refusal"):ab (Word variations have been searched)

#12 #9 OR #10 OR #11

#13 MeSH descriptor: [Child] explode all trees

#14 MeSH descriptor: [Adolescent] explode all trees

#15 (adolesc* or child* or boy* or girl* or juvenil* or minors or paediatri* or pediatri* or pubescen* or school* or student* or teen* or young or youth* or class* or preschool or pre-school):ab(Word variations have been searched)

#16 (adolesc* or child* or boy* or girl* or juvenil* or minors or paediatri* or pediatri* or pubescen* or school* or student* or teen* or young or youth* or class* or preschool or pre-school):ti(Word variations have been searched)

#17 #13 OR #14 OR #15 OR #16

#18 #8 AND #12 AND #17

**Embase**

#1 'cognitive behavioral therapy'/exp OR 'cognitive behavioral therapy'

#2 behavio*:ti AND cogniti*:ti AND therapy:ti OR cbt:ti

#3 behavio*:ab AND cogniti*:ab AND therapy:ab OR cbt:ab

#4 #1 OR #2 OR #3

#5 individual:ab AND group:ab

#6 individual:ti AND group:ti

#7 #5 OR #6

#8 #4 AND #7

#9 'anxiety disorder'/exp OR 'anxiety disorder'

#10 anxiety:ti OR anxious:ti OR phobic:ti OR fear:ti OR fears:ti OR phobia:ti OR phobias:ti OR 'panic disorder*':ti OR 'overanxious disorder*':ti OR 'avoidant disorder*':ti OR agoraphobia:ti OR 'selective mutism':ti OR 'panic attack':ti OR 'combat disorder*':ti OR 'mixed disorder*':ti OR neurosis:ti OR neuroses:ti OR neurotic:ti OR 'school refusal':ti

#11 anxiety:ab OR anxious:ab OR phobic:ab OR fear:ab OR fears:ab OR phobia:ab OR phobias:ab OR 'panic disorder*':ab OR 'overanxious disorder*':ab OR 'avoidant disorder*':ab OR agoraphobia:ab OR 'selective mutism':ab OR 'panic attack':ab OR 'combat disorder*':ab OR 'mixed disorder*':ab OR neurosis:ab OR neuroses:ab OR neurotic:ab OR 'school refusal':ab

#12 #9 OR #10 OR #11

#13 'child'/exp OR 'child'

#14 'adolescent'/exp OR 'adolescent'

#15 adolesc*:ti OR child*:ti OR boy*:ti OR girl*:ti OR juvenil*:ti OR minors:ti OR paediatri*:ti OR pediatri*:ti OR pubescen*:ti OR school*:ti OR student*:ti OR teen*:ti OR young:ti OR youth*:ti OR class*:ti OR preschool:ti OR 'pre school':ti

#16 adolesc*:ab OR child*:ab OR boy*:ab OR girl*:ab OR juvenil*:ab OR minors:ab OR paediatri*:ab OR pediatri*:ab OR pubescen*:ab OR school*:ab OR student*:ab OR teen*:ab OR young:ab OR youth*:ab OR class*:ab OR preschool:ab OR 'pre school':ab

#17 #13 OR #14 OR #15 OR #16

#18 #8 AND #12 AND #17

**PsycINFO**

#1 TI (behavio* AND cogniti* AND therapy) OR AB (behavio* AND cogniti* AND therapy)

#2 TI (CBT OR “Cognitive behavior therapy” OR “Cognitive behavioral therapy” OR “Cognitive behaviour therapy” OR “Cognitive behavioural therapy” OR “Cognitive-behavioural therapy” OR “Cognitive-behavioual therapy) OR AB (CBT OR “Cognitive behavior therapy” OR “Cognitive behavioral therapy” OR “Cognitive behaviour therapy” OR “Cognitive behavioural therapy” OR “Cognitive-behavioural therapy” OR “Cognitive-behavioual therapy”)

#3 S1 OR S2

#4 TI (group AND individual) OR AB (group AND individual)

#5 S3 AND S4

#6 SU Anxiety Disorders

#7 TI (anxiety OR anxious OR phobic OR fear OR fears OR phobia OR phobias OR “panic disorder*” OR “overanxious disorder*” OR “avoidant disorder*” OR agoraphobia OR “selective mutism” OR “panic attack speciﬁer” OR “combat disorder*” OR “mixed disorder*” OR neurosis OR neuroses OR neurotic OR “school refusal”) OR AB (anxiety OR anxious OR phobic OR fear OR fears OR phobia OR phobias OR “panic disorder*” OR “overanxious disorder*” OR “avoidant disorder*” OR agoraphobia OR “selective mutism” OR “panic attack speciﬁer” OR “combat disorder*” OR “mixed disorder*” OR neurosis OR neuroses OR neurotic OR “school refusal”)

#8 S6 OR S7

#9 TI (adolesc* OR child* OR boy* OR girl* OR juvenil* OR minors OR paediatri* OR pediatri* OR pubescen* OR school* OR student* OR teen* OR young OR youth* OR class* OR preschool OR pre-school) OR AB (adolesc* OR child* OR boy* OR girl* OR juvenil* OR minors OR paediatri* OR pediatri* OR pubescen* OR school* OR student* OR teen* OR young OR youth* OR class* OR preschool OR pre-school)

#10 SU child OR SU adolescent

#11 S9 OR S10

#12 S5 AND S8 AND S11

**Web of Science**

#1 TS=(behavio* AND cogniti* AND therapy) Indexes=SCI-EXPANDED, SSCI, A&HCI, CPCI-S, CPCI-SSH, BKCI-S, BKCI-SSH, ESCI, CCR-EXPANDED, IC Timespan=All years

#2 TS=(CBT OR “Cognitive behavior therapy” OR “Cognitive behavioral therapy” OR “Cognitive behaviour therapy” OR “Cognitive behavioural therapy” OR “Cognitive-behavioural therapy” OR “Cognitive-behavioral therapy”) Indexes=SCI-EXPANDED, SSCI, A&HCI, CPCI-S, CPCI-SSH, BKCI-S, BKCI-SSH, ESCI, CCR-EXPANDED, IC Timespan=All years

#3 #2 OR #1 Indexes=SCI-EXPANDED, SSCI, A&HCI, CPCI-S, CPCI-SSH, BKCI-S, BKCI-SSH, ESCI, CCR-EXPANDED, IC Timespan=All years

#4 TS=(group AND individual) Indexes=SCI-EXPANDED, SSCI, A&HCI, CPCI-S, CPCI-SSH, BKCI-S, BKCI-SSH, ESCI, CCR-EXPANDED, IC Timespan=All years

#5 #4 AND #3 Indexes=SCI-EXPANDED, SSCI, A&HCI, CPCI-S, CPCI-SSH, BKCI-S, BKCI-SSH, ESCI, CCR-EXPANDED, IC Timespan=All years

#6 TS=(anxiety OR anxious OR phobic OR fear OR fears OR phobia OR phobias OR "panic disorder*" OR "overanxious disorder*" OR "avoidant disorder*" OR agoraphobia OR "selective mutism" OR "panic attack specifier" OR "combat disorder*" OR "mixed disorder*" OR neurosis OR neuroses OR neurotic OR "school refusal") Indexes=SCI-EXPANDED, SSCI, A&HCI, CPCI-S, CPCI-SSH, BKCI-S, BKCI-SSH, ESCI, CCR-EXPANDED, IC Timespan=All years

#7 TS=(adolesc* OR child* OR boy* OR girl* OR juvenil* OR minors OR paediatri* OR pediatri* OR pubescen* OR school* OR student* OR teen* OR young OR youth* OR class* OR preschool OR pre-school) Indexes=SCI-EXPANDED, SSCI, A&HCI, CPCI-S, CPCI-SSH, BKCI-S, BKCI-SSH, ESCI, CCR-EXPANDED, IC Timespan=All years

#8 TS=(random* OR allocate* OR assign* OR “cross over*” OR crossover* OR controlled) Indexes=SCI-EXPANDED, SSCI, A&HCI, CPCI-S, CPCI-SSH, BKCI-S, BKCI-SSH, ESCI, CCR-EXPANDED, IC Timespan=All years

#9 #8 AND #7 AND #6 AND #5 Indexes=SCI-EXPANDED, SSCI, A&HCI, CPCI-S, CPCI-SSH, BKCI-S, BKCI-SSH, ESCI, CCR-EXPANDED, IC Timespan=All years

**CINAHL**

S1 TI (behavio* AND cogniti* AND therapy) OR AB (behavio* AND cogniti* AND therapy)

S2 TI (CBT OR “Cognitive behavior therapy” OR “Cognitive behavioral therapy” OR “Cognitive behaviour therapy” OR “Cognitive behavioural therapy” OR “Cognitive-behavioural therapy” OR “Cognitive-behavioual therapy”) OR AB (CBT OR “Cognitive behavior therapy” OR “Cognitive behavioral therapy” OR “Cognitive behaviour therapy” OR “Cognitive behavioural therapy” OR “Cognitive-behavioural therapy” OR “Cognitive-behavioual therapy”)

S3 S1 or S2

S4 TI (group AND individual) OR AB (group AND individual)

S5 S3 AND S4

S6 MH Anxiety Disorders

S7 TI (anxiety OR anxious OR phobic OR fear OR fears OR phobia OR phobias OR “panic disorder*” OR “overanxious disorder*” OR “avoidant disorder*” OR agoraphobia OR “selective mutism” OR “panic attack speciﬁer”OR “combat disorder*” OR “mixed disorder*” OR neurosisOR neuroses OR neurotic OR “school refusal”) OR AB (anxiety OR anxious OR phobic OR fear OR fears OR phobia OR phobias OR “panic disorder*” OR “overanxious disorder*” OR “avoidant disorder*” OR agoraphobia OR “selective mutism” OR “panic attack speciﬁer”OR “combat disorder*” OR “mixed disorder*” OR neurosisOR neuroses OR neurotic OR “school refusal”)

S8 S6 OR S7

S9 TI (adolesc* OR child* OR boy* OR girl* OR juvenil* OR minors OR paediatri* OR pediatri* OR pubescen* OR school* OR student* OR teen* OR young OR youth* OR class* OR preschool OR pre-school) OR AB (adolesc* OR child* OR boy* OR girl* OR juvenil* OR minors OR paediatri* OR pediatri* OR pubescen* OR school* OR student* OR teen* OR young OR youth* OR class* OR preschool OR pre-school)

S10 MM Child OR MM Adolescent

S11 S9 OR S10

S12 S5 AND S8 AND S11

**LILACS**

S1 (Anxiety Disorders or anxiety or anxious or phobic or fear or fears or phobia or phobias or “panic disorder*” or “overanxious disorder*” or “avoidant disorder*” or agoraphobia or “selective mutism” or “panic attack speciﬁer” or “combat disorder*” or “mixed disorder*” or neurosis or neuroses or neurotic or “school refusal”) and (behavio$ or cogniti$ or therapy or behavio$ and cogniti$ and therapy or CBT or “Cognitive behavior therapy” or “Cognitive behavioral therapy” or “Cognitive behaviour therapy” or “Cognitive behavioural therapy” or “Cognitive-behavioural therapy” or “Cognitive-behavioual therapy”) and (group AND individual) and (adolesc$ or child$ or boy$ or girl$ or juvenil$ or minors or paediatri$ or pediatri$ or pubescen$ or school$ or student$ or teen$ or young or youth$ or class$ or preschool or pre-school) [Title words] or (Anxiety Disorders or anxiety or anxious or phobic or fear or fears or phobia or phobias or “panic disorder*” or “overanxious disorder*” or “avoidant disorder*” or agoraphobia or “selective mutism” or “panic attack speciﬁer” or “combat disorder*” or “mixed disorder*” or neurosis or neuroses or neurotic or “school refusal”) and (behavio$ or cogniti$ or therapy or behavio$ and cogniti$ and therapy or CBT or “Cognitive behavior therapy” or “Cognitive behavioral therapy” or “Cognitive behaviour therapy” or “Cognitive behavioural therapy” or “Cognitive-behavioural therapy” or “Cognitive-behavioual therapy”) and (group AND individual) and (adolesc$ or child$ or boy$ or girl$ or juvenil$ or minors or paediatri$ or pediatri$ or pubescen$ or school$ or student$ or teen$ or young or youth$ or class$ or preschool or pre-school) [Abstract words]

**ProQuest Dissertations**

S1 bisac(Anxiety Disorders) OR ti(anxiety OR anxious OR phobic OR fear OR fears OR phobia OR phobias OR “panic disorder*” OR “overanxious disorder*” OR “avoidant disorder*” OR agoraphobia OR “selective mutism” OR “panic attack speciﬁer” OR “combat disorder*” OR “mixed disorder*” OR neurosis OR neuroses OR neurotic OR “school refusal”) OR ab(anxiety OR anxious OR phobic OR fear OR fears OR phobia OR phobias OR “panic disorder*” OR “overanxious disorder*” OR “avoidant disorder*” OR agoraphobia OR “selective mutism” OR “panic attack speciﬁer” OR “combat disorder*” OR “mixed disorder*” OR neurosis OR neuroses OR neurotic OR “school refusal”)

S2 ti(behavio* AND cogniti* AND therapy) OR ab(behavio* AND cogniti* AND therapy)

S3 ti(CBT OR “Cognitive behavior therapy” OR “Cognitive behavioral therapy” OR “Cognitive behaviour therapy” OR “Cognitive behavioural therapy” OR “Cognitive-behavioural therapy” OR “Cognitive-behavioual therapy”) OR ab(CBT OR “Cognitive behavior therapy” OR “Cognitive behavioral therapy” OR “Cognitive behaviour therapy” OR “Cognitive behavioural therapy” OR “Cognitive-behavioural therapy” OR “Cognitive-behavioual therapy”)

S4 S2 or S3

S5 ti(“group ” AND “individual ”) OR ab(“group ” AND “individual ”)

S6 S4 AND S5

S7 ti(adolesc* OR child* OR boy* OR girl* OR juvenil* OR minors OR paediatri* OR pediatri* OR pubescen* OR school* OR student* OR teen* OR young OR youth* OR class* OR preschool OR pre-school) OR ab(adolesc* OR child* OR boy* OR girl* OR juvenil* OR minors OR paediatri* OR pediatri* OR pubescen* OR school* OR student* OR teen* OR young OR youth* OR class* OR preschool OR pre-school) OR mainsubject("Child" OR "Adolescent")

S9 S1 AND S6 AND S7

**Figure S1.** Funnel plot for primary efficacy outcomes: the standardized mean difference for changed scores in anxiety disorder rating scales at post-treatment **(A)** and acceptability **(B)**.

**A**


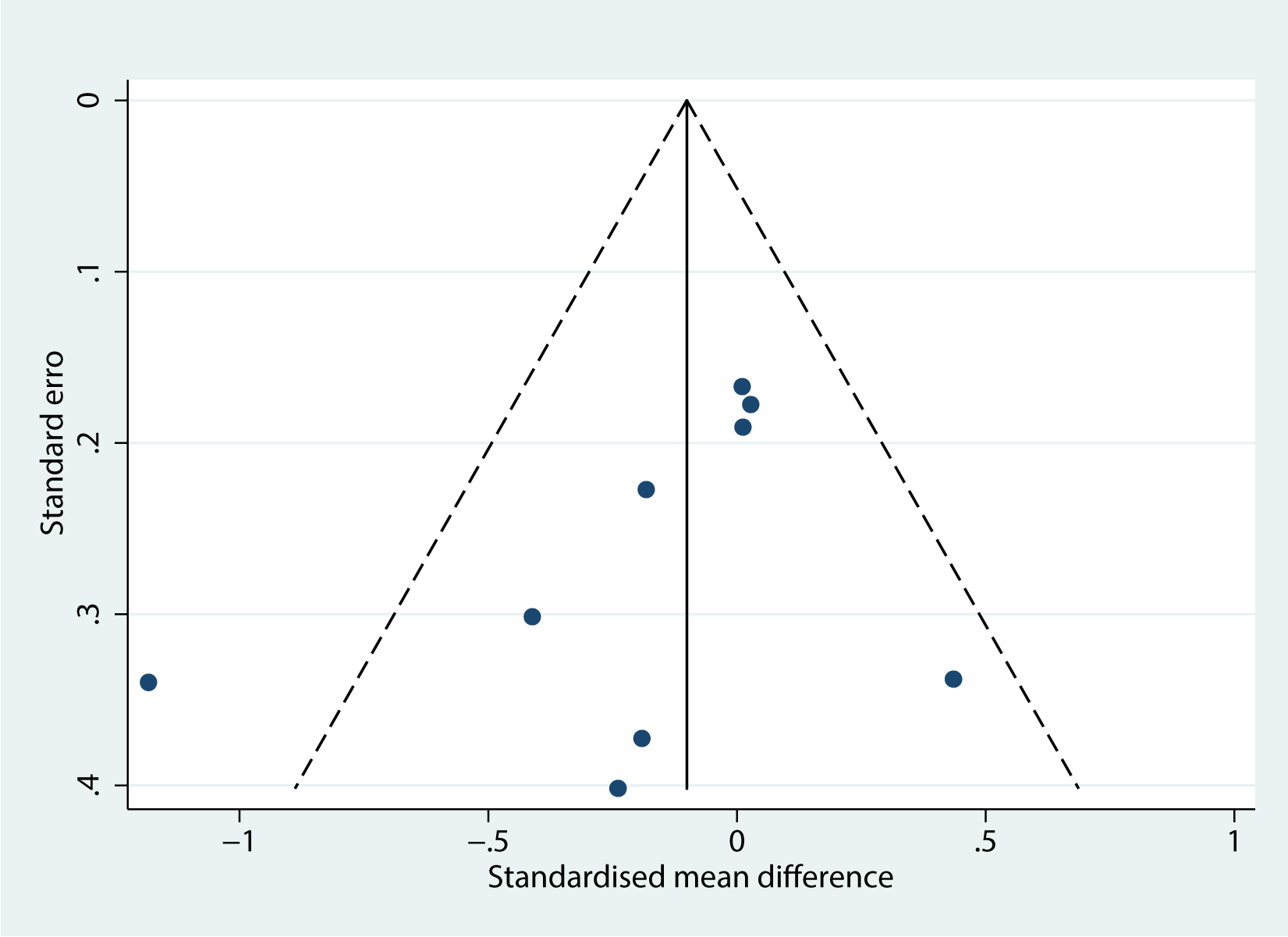


**B**


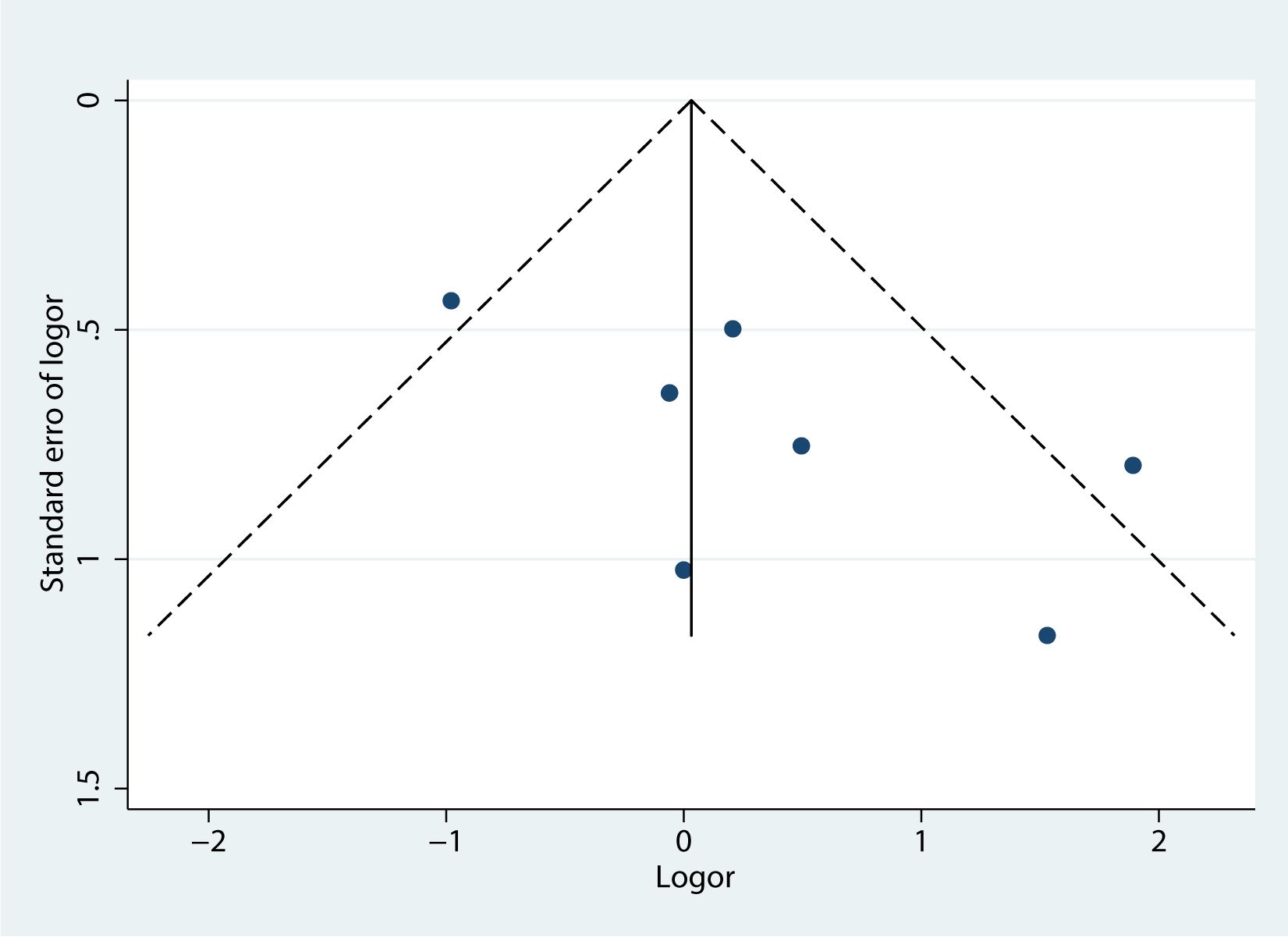

Supplement: Supplementary file 1 [file Data_Sheet_1.doc]
